# Soybean allergy in a population with a low prevalence of betulacae pollen allergy and a high soybean consumption

**DOI:** 10.1186/2045-7022-1-S1-P89

**Published:** 2011-08-12

**Authors:** Yuma Fukutomi, Sigrid Sjölander, Magnus Borres, Takuya Nakazawa, Toyota Ishii, Satoshi Nakayama, Akira Tanaka, Masami Taniguchi, Akemi Saito, Hiroshi Yasueda, Hiroyuki Nakamura, Kazuo Akiyama

**Affiliations:** 1 Sagamihara National Hospital, Clinical Research Center for Allergy and Rheumatology, Sagamihara Kanagawa, Japan; 2 Phadia AB, Uppsala, Sweden; 3 Göteborg University, Department of Pediatrics, Sahlgrenska Academy, Göteborg, Sweden; 4 Phadia KK, Tokyo, Japan; 5 Graduate School of Medical Science, Kanazawa University, Department of Environmental and Preventive Medicine, Kanazawa Ishikawa, Japan

## Background

Recent evidence has shown that birch pollen-related soybean allergy mediated by Gly m 4 is common in central Europe. However, the impact of sensitization to Gly m 4 in soybean allergic patients in a population with a low prevalence of Betulaceae pollen allergy and a high soybean consumption is unknown.

## Methods

The aim of this study was to elucidate the prevalence of sensitization to rGly m 4 in adults with soybean allergy, and to analyze the diagnostic efficiency of the IgE antibody to rGly m 4 (ImmunoCAP®) in soybean allergy in central Japan. Twenty-one soybean-allergic patients were prospectively recruited from Jan. to Dec. 2009, and their levels of IgE antibody to rGly m 4 were compared with those of general alder pollen-allergic control subjects without soybean allergy (n=85).

## Results

Although sensitization to alder pollen was not prevalent in the general outpatients of allergy departments, all the soybean–allergic patients were sensitized to alder pollen and rGly m 4. Sixty-two percent of the general alder pollen-allergic control subjects were also sensitized to rGly m 4. However, the levels of IgE antibody to rGly m 4 in soybean-allergic patients were markedly higher than those in alder pollen-allergic control subjects. The area under the receiver-operating characteristics curve for levels of IgE antibody to rGly m 4 in the diagnosis of soybean allergy was 0.86, which was significantly higher than that to the natural soybean extracts.

## Conclusion

A strong relationship between adult soybean allergy and sensitization to rGly m 4 was also observed in this population with high soybean consumption. The level of IgE antibody to CAP-rGly m 4 was an effective tool in discriminating soybean allergy from general alder pollen-allergy. This result highlights the impact of respiratory allergy to pollen-derived cross-reactive allergens on the epidemics of adult plant food allergy.

